# Audencel Immunotherapy Based on Dendritic Cells Has No Effect on Overall and Progression-Free Survival in Newly Diagnosed Glioblastoma: A Phase II Randomized Trial

**DOI:** 10.3390/cancers10100372

**Published:** 2018-10-05

**Authors:** Johanna Buchroithner, Friedrich Erhart, Josef Pichler, Georg Widhalm, Matthias Preusser, Günther Stockhammer, Martha Nowosielski, Sarah Iglseder, Christian F. Freyschlag, Stefan Oberndorfer, Karin Bordihn, Gord von Campe, Markus Hoffermann, Reinhard Ruckser, Karl Rössler, Sabine Spiegl-Kreinecker, Michael B. Fischer, Thomas Czech, Carmen Visus, Günther Krumpl, Thomas Felzmann, Christine Marosi

**Affiliations:** 1 University Clinic for Neurosurgery, Kepler University Hospital, Johannes Kepler University, Wagner-Jauregg-Weg 15, 4020 Linz, Austria; johanna.buchroithner@kepleruniklinikum.at (J.B.); Sabine.Spiegl-Kreinecker@kepleruniklinikum.at (S.S.-K.); 2 Department of Neurosurgery, Medical University of Vienna, Währinger Gürtel 18-20, 1090 Vienna, Austria; friedrich.erhart@meduniwien.ac.at (F.E.); georg.widhalm@meduniwien.ac.at (G.W.); thomas.czech@meduniwien.ac.at (T.C.); 3 Institute of Neurology, Medical University of Vienna, Währinger Gürtel 18-20, 1090 Vienna, Austria; 4 Department of Internal Medicine and Neurooncology, Kepler University Hospital, Johannes Kepler University, Wagner-Jauregg-Weg 15, 4020 Linz, Austria; Josef.Pichler@kepleruniklinikum.at; 5 Clinical Division of Medical Oncology, Department for Internal Medicine I, Medical University of Vienna, Währinger Gürtel 18-20, 1090 Vienna, Austria; Matthias.preusser@meduniwien.ac.at; 6 Department of Neurology, Medical University of Innsbruck, Christoph-Probst-Platz 1, Innrain 52, 6020 Innsbruck, Austria; guenther.stockhammer@i-med.ac.at (G.S.); Martha.Nowosielski@i-med.ac.at (M.N.); sarah.iglseder@tirol-kliniken.at (S.I.); 7 Department of Neurosurgery, Medical University of Innsbruck, Christoph-Probst-Platz 1, Innrain 52, 6020 Innsbruck, Austria; christian.freyschlag@i-med.ac.at; 8 Department of Neurology, University Clinic St. Pölten, Karl Landsteiner Privat Universität, Dunant-Platz 1, 3100 St. Pölten, Austria; Stefan.Oberndorfer@stpoelten.lknoe.at; 9 Department of Neurosurgery, Landeskrankenhaus Salzburg, University Clinic of the Paracelsus Private Medical University, Müllner Hauptstraße 48, 5020 Salzburg, Austria; k.bordihn@salk.at; 10 Department of Neurosurgery, Medical University of Graz, Auenbruggerplatz 29, 8036 Graz, Austria; gord.von-campe@medunigraz.at (G.v.C.); markus.hoffermann@lkhf.at (M.H.); 11 Department of Internal Medicine 2, Donauspital, Langobardenstraße 122, 1220 Vienna, Austria; reinhard.ruckser@aon.at; 12 Department of Neurosurgery, Friedrich-Alexander-Universität Erlangen-Nürnberg, Schwabachanlage 6, 91054 Erlangen, Germany; Karl.roessler@uk.erlangen.de; 13 Department of Blood Group Serology and Transfusion Medicine, Medical University of Vienna, Währinger Gürtel 18-20, 1090 Vienna, Austria; michael.b.fischer@meduniwien.ac.at; 14 Activartis Biotech GmbH, Wilhelminenstraße 91/IIf, 1160 Vienna, Austria; maria-carmen.visus-miguel@aoporphan.com (C.V.); g.krumpl@medresnet.com (G.K.); tfelzm@gmail.com (T.F.)

**Keywords:** glioblastoma, active immunotherapy, dendritic cells, tumor vaccine, phase II, randomized, clinical trial

## Abstract

Dendritic cells (DCs) are antigen-presenting cells that are capable of priming anti-tumor immune responses, thus serving as attractive tools to generate tumor vaccines. In this multicentric randomized open-label phase II study, we investigated the efficacy of vaccination with tumor lysate-charged autologous DCs (Audencel) in newly diagnosed glioblastoma multiforme (GBM). Patients aged 18 to 70 years with histologically proven primary GBM and resection of at least 70% were randomized 1:1 to standard of care (SOC) or SOC plus vaccination (weekly intranodal application in weeks seven to 10, followed by monthly intervals). The primary endpoint was progression-free survival at 12 months. Secondary endpoints were overall survival, safety, and toxicity. Seventy-six adult patients were analyzed in this study. Vaccinations were given for seven (3–20) months on average. No severe toxicity was attributable to vaccination. Seven patients showed flu-like symptoms, and six patients developed local skin reactions. Progression-free survival at 12 months did not differ significantly between the control and vaccine groups (28.4% versus 24.5%, *p* = 0.9975). Median overall survival was similar with 18.3 months (vaccine: 564 days, 95% CI: 436–671 versus control: 568 days, 95% CI: 349–680; *p* = 0.89, harzard ratio (HR) 0.99). Hence, in this trial, the clinical outcomes of patients with primary GBM could not be improved by the addition of Audencel to SOC.

## 1. Introduction

Patients with glioblastoma multiforme (GBM) represent an unmet need for effective treatment. Current standard of care (SOC) achieves a rate of 27.2% of patients alive after two years, with radiotherapy and concomitant adjuvant temozolomide (TMZ) [1]. The results of recent trials showed that after 24 months, 12.5% to 45% of newly diagnosed GBM patients were still alive, e.g., 12.5% of patients with unmethylated O-6-methylguanine-DNA methyltransferase (MGMT) promoters treated with cilengitide in addition to SOC [2], as compared to 45% of patients with methylated MGMT promoter [3]. In two trials using bevacizumab in addition to SOC, 16.1% and 25.4% of enrolled patients survived for two years [4,5] and 15% survived with dose-dense TMZ [6]. Recently, the phase III trial using rindopepimut, a vaccine against the mutated form of the epithelial growth factor receptor variant III (EGFRvIII), was stopped because of futility. In all of these trials, no significant improvement compared to the SOC was obtained. In contrast, the most recent trial used a device generating electromagnetic fields to the tumor bed for maintenance therapy after concomitant chemoradiation therapy [7], and slightly more than 30% of patients survived for two years. These results demonstrate the great need to develop novel effective therapies against malignant gliomas. Most recently, immunotherapies have shown activity in a number of various cancers, including hematological diseases and solid tumors, in particular malignant melanoma, renal carcinoma, and non-small cell lung cancer. In 2010, Sipuleucel-T was the first dendritic cell (DC)-based vaccine approved by the US Food and Drug Administration (FDA). It has shown consistently significant survival benefit in patients with castrate resistant prostate cancer. Most of the benefit was observed in patients with low tumor burden [8,9,10,11]. Also, against GBM, a number of DC-based therapeutic vaccines are currently being developed [12]. So far, clinical trials indicated the safety and feasibility of DC vaccines against GBM [12,13,14]. Despite recent promising interim results from a phase III trial, efficacy has not been shown yet [15].

The brain has unique features in interaction with the immune system, whose structures and functions have been discovered only recently [16,17,18]. GBM is known to grow in a highly immunosuppressive microenvironment that is favored by tumor hypoxia and the presence of immunosuppressive cytokines produced partly by the glioma cells, but also by the immune system [19]. Tumor-infiltrating immune cells e.g., by T helper cells and cytotoxic T cells, DCs, monocytes, and macrophages [20] may make up 15% of the tumor mass. Macrophages infiltrating the tumor bulk are mainly of the immunosuppressive M2 phenotype [21,22]. The hypoxic environment stimulates the activation of signal transducers and activators of transcription 3 (STAT 3), which induce the formation of tumor-associated macrophages (TAMS), resulting in neoangiogenesis and the invasive growth of GBMs [23]. Within the glioma microenvironment, antigen presentation by microglia is compromised. Moreover, in the systemic circulation, patients show a decreased proportion of CD4 positive T cells and an increased proportion of CD8 positive T cells in peripheral blood, illustrating their vulnerability to nosocomial infections. This shortened overview lists only partly the mechanisms by which gliomas escape immunosurveillance and promote the survival of tumor cells.

DCs are antigen-presenting cells that guide immune reactions [24,25]. Contact with pathogen-associated microbial pattern molecules, which are also known as “danger molecules”, such as lipopolysaccharides (LPS), switches DCs into a potent immune stimulatory mode of action, “maturation”, characterized by the release of interleukin (IL)-12 [26,27,28]. This distinguishes “Audencel”, the DC-based cancer immunotherapy technology (CIT) that is used in this study, from other DC-CIT concepts [29,30]. IL-12-secreting DCs trigger robust helper T-lymphocyte type 1 and cytotoxic T-lymphocyte dominated immune responses in vitro [30,31,32] as well as in vivo [29,33]. This unique immunostimulatory capability of DCs has been investigated as a way to combat cancers through counteracting their immunosuppressive features for several years [31,34,35].

We report on a randomized controlled phase II trial with a fixed starting point for vaccination therapy within one week after the end of chemoradiation, an active immunotherapy based on autologous DCs charged with autologous tumor lysate (“Audencel”) for patients with newly diagnosed GBM in addition to standard radiochemotherapy treatment.

## 2. Results

### 2.1. Survival Outcome

A total of 76 patients were enrolled and analyzed in this study: 34 in the experimental arm with Audencel, and 42 in the control arm. Initially, after treatment center-specific 1:1 randomization, 39 patients had been formally included in the experimental arm and 42 had been formally included in the control arm. Five patients of the experimental arm were later excluded because they had been double-counted (due to switching from one treatment center to another) or because they did not actually meet the formal inclusion criteria (e.g., age). As a result, the experimental cohort for analysis consisted of 34 patients. Mutations in the isocitrate dehydrogenase 1 (IDH1) gene were not detected in any patient, confirming that all of the patients suffered from primary GBM. Their baseline characteristics and demographics are given in Table 1. Twenty-five female and 51 male patients entered the study, aged from 19 to 70 years, with a median age of 54 years. The median duration from surgery to the start of radiochemotherapy was four weeks in both groups.

**Figure 1:**
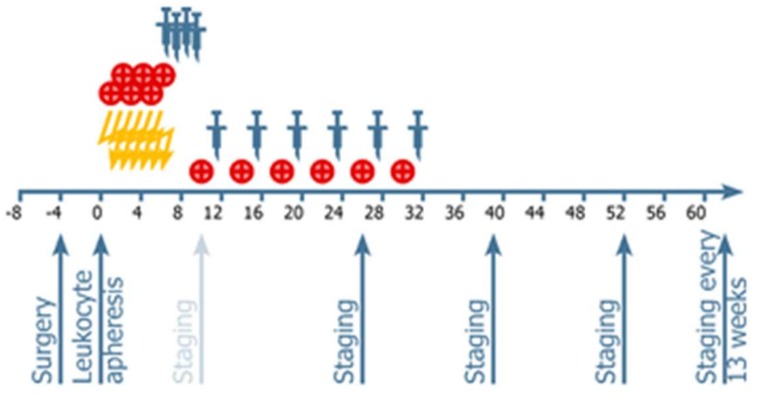
Treatment schedule.

Patients randomized to the vaccination arm underwent leucocyte apheresis before the start of radiotherapy, and subsequently received vaccine treatment by intranodal application starting in week 7 after the treatment start, as detailed previously (Figure 1). The duration of vaccination was seven months on average (3–20 months). No patient stopped vaccination due to side effects. 

All of the patients were analyzed according to intention-to-treat. The main reason for treatment discontinuation was disease progression in both arms. One female patient aged 65 years and randomized to the experimental arm experienced status epilepticus on the day after leucocyte apheresis; she was admitted to the hospital where the status was terminated, but disease progression was diagnosed. She died without receiving postsurgical anti-tumor treatment. All of the other randomized patients could be treated according to the SOC, all of the patients were able to receive radiotherapy up to 60 gray (Gy), and concomitant and adjuvant therapy with TMZ, respectively.

Patients randomized into the experimental arm received four to 15 vaccinations, with 10 being the average.

**Figure 2:**
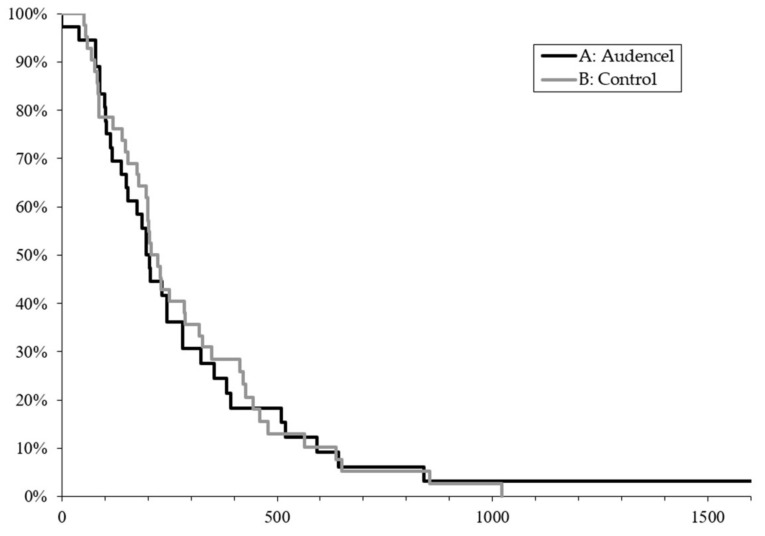
Progression-free survival in days. Kaplan–Meier analysis by treatment group indicates no difference between the vaccine and the control arm (*p* = 0.83).

Time to progression was between six and eight months, e.g., 204 days, 95% CI: (138; 280) in the vaccine arm versus 6.9 months e.g., 210 days, 95% CI (179; 286) (*p* = 0.83) in the control arm, as shown in Figure 2. Pseudoprogression occurred in three patients of the Audencel group and eight patients of the control group. For details regarding the definition of progression and pseudoprogression, see the Materials and Methods section.

**Figure 3:**
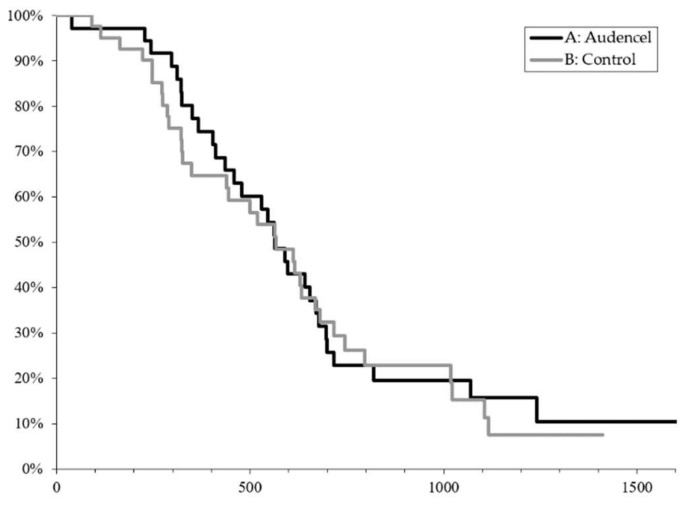
Overall survival in days. Kaplan–Meier analysis by treatment group indicates no difference between the vaccine and the control arm (*p* = 0.99).

Overall, survival was similar in both groups: vaccination group: 564 days, 95% CI: (436; 671) versus control group: 568 days, 95% CI (349; 680) (*p* = 0.99, Figure 3). The survival of patients with methylated MGMT promoter was 800 days on average, whereas it reached 340 days in patients who had the unmethylated MGMT promoter, irrespective of whether patients received an add-on vaccination or not (for details on MGMT, see below). One vaccinated patient survived beyond five years.

### 2.2. Adverse Events

There were more adverse events recorded in the treatment group as compared to the control group; also, events related to the alkylating drug TMZ were more frequently observed in the Audencel group than in the control group. However, except for severe thrombocytopenia, these differences did not reach significance. There was no increase of intracerebral bleedings observed in the vaccine group (Table 2).

Adverse events that were related to the vaccine therapy consisted mainly of local pain and local reactions; these were mostly mild to moderate, and affected six out of 34 patients (18%). Seven vaccinated patients reacted with fever up to 39 °C and general weakness and joint pain on the day of vaccine administration, which resolved spontaneously within 12 h. Of note, there were no signs of autoimmune toxicity in the central nervous system (CNS) observed in vaccinated patients.

### 2.3. Treatment after Relapse

Six patients in the Audencel group and 12 patients in the control group received no active treatment in relapse. Four patients in the Audencel group and three patients in the control group underwent a second surgical resection in relapse.

The most common treatment for relapse consisted of bevacizumab (10 mg/kg, given every two weeks) administered to 20 patients in the Audencel group and 23 in the control group, followed by TMZ rechallenge (nine patients in the Audencel group and 12 patients in the control group).

### 2.4. Extent of Resection 

In glioblastoma, the extent of resection (EOR) is known to have an influence on survival [36]. In the specific context of DC immunotherapy, it has been discussed for a number of malignant entities that DC immunotherapy might be most applicable to patients with minimal residual disease [37,38]. Thus, we investigated a potential influence of EOR on outcome in our clinical trial. Patients of the Audencel and the SOC cohort were stratified into three groups, with each based on EOR (gross total resection of 100% versus partial resection of 90–99% versus partial resection of 70–90%). No patient was below the threshold of at least 70% EOR, as this was an inclusion criterion. Exploratory Kaplan–Meier analysis showed that EOR did not have an influence on the OS outcome of the Audencel cohort compared to the SOC cohort for all three groups (*p* = 0.762, Figure S1).

### 2.5. DC Vaccine Quality

DC vaccine quality was ensured via stringent phenotypical and functional release criteria for each vaccine batch produced (for details, see the Supplementary Material). Functionally, interleukin-12 (IL-12) production capacity and T-cell stimulation capacity (via mixed leukocyte reactions, MLR) were measured in analogy to the DC trials performed by others [39,40]. Release criteria were >100 pg/mL IL-12, and at least 30% T-cell proliferation upon DC co-culture in relation to reference stimulation with staphylococcal enterotoxin A/B (SEA/SEB). These criteria ensured standard quality for all of the batches, but still left room for variation. Thus, we tested whether vaccine quality had a relation to outcome. Exploratory Pearson correlation analysis showed that neither IL-12 production (*p* = 0.891, Figure S2) nor T-cell stimulation capacity at various DC:T-cell ratios (1:5 *p* = 0.603, 1:10 *p* = 0.488, 1:20 *p* = 0.330, Figure S2) had an impact on survival (OS).

### 2.6. DC Vaccine Quantity 

As mentioned, depending on their individual course of disease, all of the Audencel-treated patients received four to 15 vaccinations, with the exception of the one patient who died prior to vaccination. While four vaccinations were the minimum, again, there was variation in the final number of vaccinations given. Hence, we also explored a potential relation of the number of vaccines received and patient outcome. In Pearson correlation analysis, a trend toward a positive association was seen, but without reaching statistical significance (*p* = 0.081, Figure S3).

### 2.7. MGMT Promoter Methylation

Thirty-seven tumor samples were available for MGMT promoter methylation testing by pyrosequencing. Out of these, 35% (13/37) harbored a methylated MGMT promoter. Over half (65%) of the tumors investigated were shown to have a mean methylation percentage below 8%, and were therefore designated as unmethylated.

**Figure 4:**
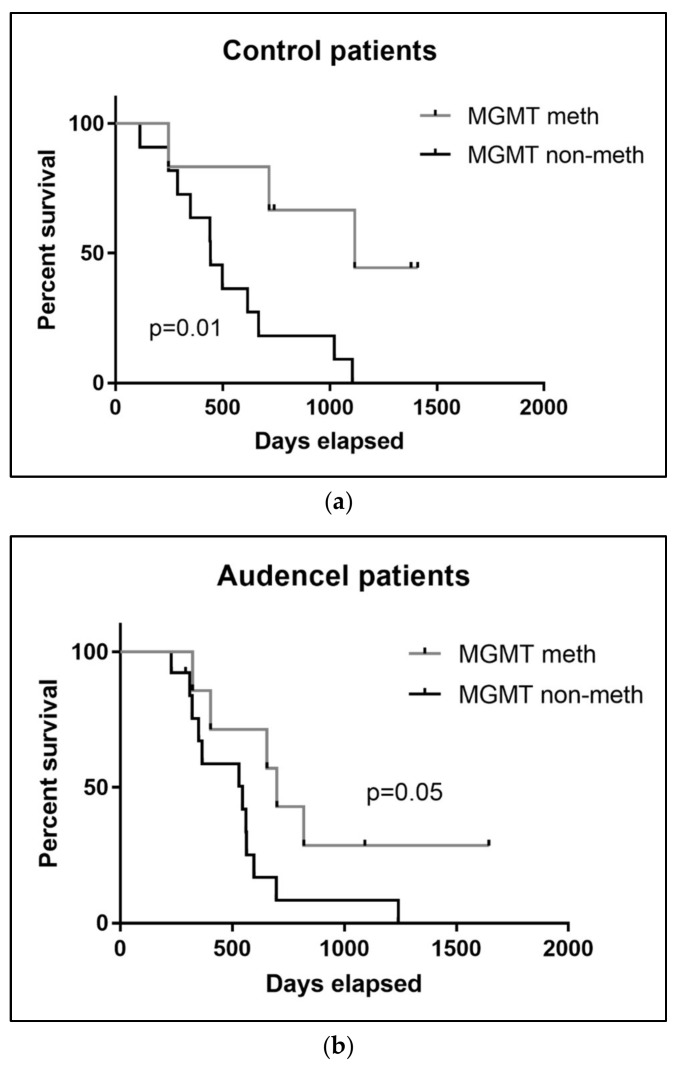
Impact of MGMT promoter methylation status on overall survival (**a**) in the control group (*p* = 0.01) and (**b**) the Audencel group (*p* = 0.05).

The fraction of patients with a methylated MGMT promoter was identical for the Audencel group (35%, seven out of 20) and the control/SOC group (35%, six out of 17). In the control/SOC group, the MGMT status had a significant impact on overall survival: control patients with a methylated MGMT promoter had a significantly better outcome (*p* = 0.01, Figure 4). For Audencel patients, a similar trend was visible; however, it did not reach statistical significance (*p* = 0.05, Figure 4). It is well-known that MGMT methylation is a predictor for the effectiveness of temozolomide chemotherapy [41], which was in line with our observation in the control/SOC group. Why the effect was not significant in the Audencel group remains to be determined. One overall caveat of the exploratory MGMT analysis presented here is the limited sample availability, as MGMT measurement was only retrospectively introduced to the study (see Section 4. Material and Methods). Validation in larger patient cohorts will be necessary.

## 3. Discussion

The addition of Audencel, an autologous DC-based vaccine with tumor peptides, to SOC in patients with newly diagnosed GBM failed to prolong progression-free survival (PFS) and OS. However, vaccinated patients developed an active immune response, as shown by the associated in vitro surveillance [42], although this was not sufficient to avoid tumor recurrence. The median OS of 18.3 months compares to the actually reported treatment results for GBM patients [4,6,7]. Of note, one of the vaccinated patients became a long-term survivor for more than five years.

Previous trials using DC-based vaccinations did not enable anticipating the lack of efficacy that was experienced in this trial. In a review published in 2013, Bregy et al. [43] evaluated 19 studies and two case reports using DC-based vaccinations for 403 glioma patients; currently, more than 20 trials are ongoing or have recently been terminated. Thirteen of the trials summarized by Bregy et al. also included patients with recurrent gliomas; nine trials included patients with WHO III gliomas, only three trials focused on patients with newly diagnosed GBM; among them was Ardon et al. with 77 patients [44]. Most notably, 11 out of 12 studies in newly diagnosed GBM showed an increase of survival duration, with seven studies reporting a median survival duration that was longer than two years [14,39,45,46,47,48,49], and one of them was even more than three years [50]. However, in most of the trials, the starting point of vaccination therapy is reported, and most noticeably, only in one study was an early start of vaccination within one to two months following surgery reported [46]. In five studies, the start of vaccination was delayed: from between six and seven weeks after the termination of radiotherapy [39], nine weeks after surgery [44], or even as long as seven to 30 weeks after surgery [14], 18 weeks (in median) after surgery [47] and even after tumor recurrence [48]. In one study, non-progressing patients after chemoradiation were included [50], which also implied a certain delay, allowing for an magnetic resonance imaging (MRI) assessment prior to vaccination start. The delay before initiating vaccination therapy avoids the inclusion of early progressing patients, and is an effective (statistical) mechanism for prolonging survival. Of note, similar to in our study, in none of these studies were autoimmune reactions reported in vaccinated patients. In one study, an inoculation of a cutaneous GBM at the site of a cutaneous injection of irradiated tumor cells for delayed-type hypersensitivity (DTH) testing was mentioned [51].

An alternative approach to the use of autologous tumor lysate to load dendritic cells is the use of selected peptide antigens, either glioma-associated antigens [52,53], or as in several ongoing trials, antigens associated with glioma stem cells [54] or mRNA derived from autologous neurospheres [40]. Furthermore, a recent phase II study using ICT-107, which is a DC vaccine of autologous DCs pulsed with 20 μg/mL of each of six synthetic peptides that are associated with glioma stem cell antigens, enrolled 124 patients with newly diagnosed GBMs. These patients were treated first with standard chemoradiation and underwent four weekly vaccinations during the following month, and further vaccinations during adjuvant treatment. There was a survival benefit in vaccinated patients with the human leukocyte antigen (HLA) haplotype A2, particularly for MGMT promoter methylated and vaccinated patients. A phase III was launched, but it was then suspended, which was apparently due to insufficient funding [12].

Several reasons may explain the failure of improving the outcome of glioblastoma patients that was observed in this study.

Firstly, the GBM tumor antigens that were presented by DCs might not have been effective triggers of an immune response to inhibit glioma growth. The vast majority of protein that is taken up, processed, and presented by the DCs might be normal, healthy, and not show any mutation that contributes to the malignant phenotype of tumor cells. Moreover, tumor lysate-loaded DCs not only contain autoantigens that are expressed by nervous tissue alone. A plethora of proteins will be the same as in any other cells, similar to the proteins that are involved in metabolism, the cytoskeleton, gene expression, and many more. Other cells in the tumor tissue will end up in DCs as well, such as blood vessel cells, and cells from the tumor’s connective tissue even infiltrating immune cells. Furthermore, not only intracellular proteins will be present in the tumor material, but also extracellular matrix proteins. Therefore, only a minority of proteins that are loaded onto DCs might have actually been immunogenic. Interestingly, in this study—similar to other DC vaccination studies reported previously—no event related to autoimmune toxicity, neither in CNS nor systemically, was observed. This can support the argument that only insufficient immune reactions were triggered by the vaccine, against both malignant as well as healthy tissue antigens. In other immunotherapeutic modalities, such as chimeric antigen receptor (CAR) T cells, fulminant immune reactions (e.g., the cytokine release syndrome) have been observed [55]. Such phenomena can be an indicator for anti-tumor efficacy. None of these fulminant reactions were observed in the present trial. As a consequence of the apparently low immunogenicity, future studies could explore an augmentation of anti-tumor immune reactions, e.g., via the combination of DC vaccination with checkpoint inhibitors.

A second potential reason for the failure of our treatment concept could be found in the hematotoxic effect of TMZ impeding the multiplication of effector T cells that should reverse the immune tolerance of the tumor. In the case of DC-CIT, the parallel application of chemotherapy might prevent an effective anti-tumor immunity from developing by killing the majority of primed cells during their proliferative phase. It is likely that this was the case in this study, which would have contributed significantly to the lack of survival benefit in patients receiving Audencel.

Thirdly, even if a number of groups are working on similar DC vaccination technologies, the production protocols are never exactly the same. One driver is the complexity of the production process that involves multiple steps in Good Manufacturing Practice (GMP) cell processing facilities. Thus, in light of encouraging prior data by other groups [14,15,47,56], the disappointing results of this trial might stem from DC production differences. For instance, DCs in this study were matured with LPS/IFNγ, while other groups do not have this step in their production protocol [47,51,56]. Future studies should compare different DC production techniques to clarify the possible relevance of technological specifics. DC production *quality per se*—following our predefined production protocols and technologies mentioned above—did not seem to be an influencing factor based on the functional DC vaccine analyses that we made (see Section 2. Results).

A fourth potential reason could be an insufficient number of DCs administered by cycle. In fact, nine out of the 12 studies that were reported by Bregy et al. and the (recently suspended) ICT-107 trial used at least twice the number of dendritic cells per dose. Based on a prior clinical study with Audencel [31], and for the regulatory reasons that resulted, less DCs per dose than those reported by others were given in this study. That quantity might generally matter is supported by our observation of a non-significant, trend-level association between the number of vaccines given and the outcome. While the number of vaccines was dependent on the individual patient’s course of disease, and can thus not be changed, future studies of the Audencel technology could consider higher doses per vaccination to increase the total quantity of DCs delivered.

## 4. Materials and Methods 

The Audencel trial was a national randomized multicenter open label phase II trial including nine active sites in Austria that was performed between 2010–2015 (first patient in April 2010, last patient out June 2015). The trial ended as planned when all of the patients reached the predefined observation period of at least 12 months (see primary objective below). Eligible patients were aged 18 to 70 years, with newly diagnosed, histologically confirmed primary GBM. Tumor samples were tested for IDH mutation and MGMT promoter methylation according to previously reported methods [57,58]—for details, see below.

Patients were eligible if their tumor had been resected for at least 70% as confirmed by a postsurgical MRI scan. Additional inclusion criteria were written informed consent, the availability of tumor tissue for vaccine preparation, as well as adequate hematological, renal, and hepatic function. Exclusion criteria were a lack of sufficient amount of tumor protein (100 µg) for Audencel production, anti-neoplastic chemotherapy or radiotherapy for four weeks before entering the study, participation in another therapeutic clinical trial, a positive pregnancy test or breast-feeding, patients unwilling to perform a safe method of birth control, and known hypersensitivity to TMZ.

The primary objective of the study was the improvement of progression-free survival (PFS) at 12 months, which was measured as the percentage of non-progressive patients one year after a post-operative MRI scan and treated according to SOC, consisting of maximal safe surgical resection, irradiation, oral chemotherapy with TMZ, and Audencel as add-on therapy, in comparison to the control group of patients receiving SOC alone. Secondary objectives included the improvement of PFS measured as the percentage of non-progressive patients with newly diagnosed GBM at 18 and 24 months. Further, median overall survival (OS), as well as quality of life (QOL), and the safety and feasibility of Audencel immunotherapy were analyzed.

The study was conducted according to the Helsinki Declaration and approved by the ethics committees of all of the participating centers (TRX 2/P-II-018). All of the patients gave oral and written informed consent. The study was registered with EudraCT number 2009-015979-27. The full trial protocol is available from the authors upon reasonable request.

### 4.1. Randomization 

Patients were stratified according to site, age, sex, and performance status and centrally randomized with the use of an interactive voice respond system in a 1:1 ratio into the treatment groups. Randomization was performed for each of the nine treatment centers individually.

### 4.2. Procedures: Treatments Administered

All of the patients were treated according to the current SOC for GBM, using conformal radiation therapy with two Gy/fraction up to 60 Gy, with concomitant treatment with 75 mg/m^2^ TMZ followed by six cycles of adjuvant TMZ at a dose of 150–200 mg/m^2^ for five days of each 28-day cycle. For safety evaluations, blood samples were taken weekly during radiochemotherapy and the following four weeks, then at the beginning of each adjuvant cycle and whenever clinically indicated. Radiation therapy had to start within four weeks after the postoperative MRI scan. For patients in the vaccine group, Audencel was produced individually as a personalized cellular medicine. Production followed prior research on Audencel [20,30,31,59]. Leucocyte apheresis had to be done before initiation of radiation therapy. Vaccine preparation and quality control are described in detail in the Supplementary Material.

### 4.3. Vaccine Administration

Audencel was administered by injection into an inguinal lymph node using visual ultrasound control. Each single dose consisted of 1–5 × 10^6^ autologous DCs in 100 µL. The rationale for this dosing regimen were regulatory (toxicity) considerations, as a prior Audencel clinical trial [34] had also used this dosage for intranodal injections, and no toxicity had been observed in that earlier study. Vaccinations were started in week seven, just after chemoradiation, with three boosting doses applied weekly to week 10, which was the start of adjuvant therapy with TMZ (Figure 1). Subsequently, vaccinations were given in monthly intervals, two weeks after start of adjuvant TMZ treatment, in weeks 12, 16, 20, 24, and 32. After the completion of six adjuvant cycles with TMZ, vaccination was pursued in a three-month interval, as long as autologous vaccine was available.

Local tolerability was assessed in all of the patients following each Audencel application. Before administering a subsequent vaccine, the injection site of the previous vaccination was inspected and assessed. Local tolerability including pain, itching, induration, edema, and erythema were evaluated. Adverse events (AEs) were recorded and graded according to CTC 4.0.

### 4.4. Neuroimaging and Response Assessment

Neuroradiological examinations were carried out using brain MRI scans with and without gadolinium enhancement before surgery and within the first 48 hours after surgery. The postoperative MRI scan represented the baseline for the evaluation of disease progression. The next follow-up MRI scan was performed 10 weeks after start of chemoradiation ± two weeks, and then every three months ± two weeks. In case of clinical deterioration, an MRI scan was performed as soon as possible to allow decisions about disease progression.

The response assessment was based on the MRI response assessment criteria that were currently valid at the time of study protocol approval, namely the MacDonald criteria [60]. According to these, progression is defined as a ≥25% increase in known contrast-enhancing lesions or any new lesion. However, prior studies taught us that a therapy-induced immune reaction might lead to inflammation and consequently to a new (therapy-induced) disruption of the blood–brain barrier with new MRI contrast enhancement. To avoid the premature discontinuation of treatment, therapy was applied according to the study protocol as long as pseudoprogression was suspected and the MRI interval was shortened. When clinical worsening occurred and the follow-up MRI showed a further increase of contrast enhancement, true tumor progression was assumed and therapy was discontinued. If the follow-up MRI showed stable or even decreasing contrast enhancement with a stable or improving clinical condition, pseudoprogression was still assumed, and treatment was continued. Progression-free survival in our study was defined as the survival from study entry until true tumor progression was confirmed by a second MRI scan (in case of suspected pseudoprogression).

### 4.5. Treatment after Relapse

Treatment after relapse was left to the investigator’s choice.

### 4.6. DNA Extraction and MGMT Promoter Methylation Analysis

In the initial clinical trial protocol that was developed in the year 2009, MGMT testing was not established as standard procedure for patients included in the trial; it was added ex post. A retrospective collection of samples for MGMT analysis was thus only successful in a fraction of patients (see Section 2. Results). Depending on availability, DNA was extracted from either tumor tissue or formalin-fixed paraffin-embedded tissue (FFPE) sections using the QiAmp DNA Mini-Kit and FFPE tissue kit (both Qiagen, Hilden, Germany) according to the manufacturer’s instructions. Subsequently, DNA modification was performed with the EpiTect Bisulfite Kit (Qiagen). Using the Therascreen MGMT Pyro Kit (Qiagen), bisulfite converted genomic DNA was amplified by PCR and sequenced on a Pyromark Q24 (Qiagen, Germany) system. Following previous research [58], a mean value of the methylation percentage of the four CpG sites investigated was calculated. Accordingly, a mean methylation >8% was considered as methylated.

### 4.7. IDH1 Mutation Analysis

IDH1 mutation status was registered via a mutation-specific antibody (Anti-IDH1 R132H) that was used for immunohistochemistry according to the previously published standard “Vienna” protocol [61].

### 4.8. Statistical Analysis

All of the statistical analyses were performed using SAS version 9.3 or GraphPad Prism 7.04.

Descriptive analyses of continuous variables (summary statistics) were described with the number of non-missing observations, arithmetic mean, standard deviation (±SD), median, quartiles (Q1 and Q3), and range (minimum and maximum). Categorical variables (frequency statistics) were described with the number of non-missing observations and percentages. Percentages were calculated within each stratum on the total number of non-missing observations.

Progression-free survival and overall survival were estimated using the Kaplan–Meier method. Treatment groups were compared using a log-rank test. A Cox proportional hazards model with stratification according to randomization strata was used for the calculation of hazard ratios and 95% confidence intervals. All of the outcome tests were done with the intention to treat the population, and safety was assessed for all patients randomized into the study.

Statistical tests were two-sided with a significance level of 5%. All of the *p*-values were rounded to four decimal places and were presented as nominal p-values with no multiplicity adjustment.

The study sample size was calculated in order to detect a doubling of surviving patients at 12 months with 80% power.

## 5. Conclusions

To summarize, the Audencel trial did not meet our expectations. However, the challenging multicentric design was feasible in terms of logistics and the practicability of delivering a personalized, custom-made cellular therapy. The vaccination was well-tolerated, and no signs of autoimmunity were detected. Vaccinated patients developed tumor directed immune responses [42], so it seems justified to further develop vaccination strategies that take into account the failure of this trial in the future speculatively by combining DC vaccination with the administration of immune check point inhibitors, and by increasing the dose or other strategies.

## Figures and Tables

**Table 1:** Patient characteristics and demographics at baseline.

Patients	Audencel Group	Control Group
N	34	42
Sex		
female	12	13
male	22	29
Age (years)	54.6	54
ECOG		
0	14 (41%)	14 (33%)
1	20 (59%)	21 (50%)
2	0	7 (17%)
>2	0	0
Surgery		
Gross total resection	24 (71%)	35 (83%)
Partial resection	10 (29%)	7 (17%)
Number of target lesions at post OP screening		
0	24 (71%)	35 (83%)
1	10 (29%)	7 (17%)
Number of non-target lesions at post-OP screening		
0	34	42
>1	0	0
Anti-epileptic drugs		
Non enzyme-inducing AEDs	11	18
Enzyme inducing AEDs	0	0
MGMT promoter methylation	*N* = 20 samples measured	*N* = 17 samples measured
methylated	7/20 35%	6/17 35%
unmethylated	13/20 65%	11/17 65%

ECOG: Eastern Cooperative Oncology Group; OP: operation; AED: anit-epileptic drugs; MGMT: O-6-methylguanine-DNA methyltransferase.

**Table 2:** Audencel toxicity assessment according to WHO guidelines.

Toxicity	Control *n* = 42					Audencel *n* = 34				
WHO	II	%	III-IV	%	All Grades	II	%	III-IV	%	All Grades
**Haematologic toxicity**										
Anemia	0	0	0	0	0	3	8.2	0	0	3
Leucopenia	3	7.1	2	5	5	2	6	2	6	4
Lymphopenia	2	4.8	0	0	2	2	6	1	3	3
Thrombopenia	9	21	2	5	11	6	17	7	20.50	13
**Non-haematologic toxicity**										
Fatigue	9	21	1	2	10	15	45	3	8	18
Headache	11	26	2	5	13	13	38	2	6	15
Nausea	7	17	0	0	7	13	38	1	3	14
Vomiting	4	9.5	0	0	4	4	12	0	0	4
VTE event	0	0	3	7	3	0	0	0	0	0
Intracranial bleeding	0	0	1	2	1	0	0	0	0	0
Rash	0	0	1	2	1	0	0	2	5	2
Influenza like illness	1	2.4	0	0	1	3	8.2	0	0	3
Fever	2	4.8	0	0	2	7	21	0	0	7
**Vaccination site reaction**	not relevant									
erythema						6	18	0	0	6
pruritus						6	18	0	0	6
Pain, induration						6	18	0	0	6

VTE: venous thromboembolism.

## Supplementary Materials

The following are available online at http://www.mdpi.com/2072-6694/10/10/372/s1, Figure S1: Influence of extent-of-resection (EOS) on overall survival (OS); Figure S2: Relation of DC vaccine quality control and overall survival (OS); Figure S3: Analysis of potential influence of number of vaccinations received on overall survival (OS); Figure S4: CONSORT 2010 Flow Diagram. Table S1: CONSORT 2010 checklist of information to include when reporting a randomised trial.
