# Supplementary material for: Audencel Immunotherapy Based on Dendritic Cells Has No Effect on Overall and Progression-Free Survival in Newly Diagnosed Glioblastoma: A Phase II Randomized Trial
Source: Cancers (Basel). 2018 Oct 5;10(10):372. doi: 10.3390/cancers10100372 (PMC6210090; doi:10.3390/cancers10100372)
Supplement: Supplementary file 1 [file cancers-10-00372-s001.pdf]

Supplementary Materials: Audencel Immunotherapy Based on Dendritic Cells Has No Effect on Overall and Progression-Free Survival in Newly Diagnosed Glioblastoma: a Phase II Randomized Trial

Johanna Buchroithner, Friedrich Erhart, Josef Pichler, Georg Widhalm, Matthias Preusser, Günther Stockhammer, Martha Nowosielski, Sarah Iglseder, Christian F. Freyschlag, Stefan Oberndorfer, Karin Bordihn, Gord von Campe, Markus Hoffermann, Reinhard Ruckser, Karl Rössler, Sabine Spiegl-Kreinecker, Michael B. Fischer, Thomas Czech, Carmen Visus, Günther Krumpl, Thomas Felzmann and Christine Marosi

Commented [FE1]: Authorship order was changed by request from the senior corresponding author – details see email

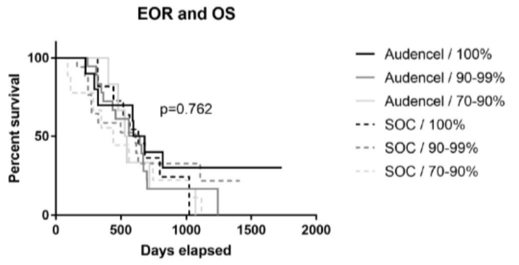

Figure S1. Influence of extent-of-resection (EOS) on overall survival (OS). Patients of the Audencel cohort and the SOC cohort were stratified into three groups based on the EOR that could be achieved (100% vs 90-99% vs. 70-90%). A minimal EOR of 70% was an inclusion criterion for the study, so no study patient was below that threshold. Kaplan-Meier analysis shows that EOR did not have an influence on the OS outcome in the Audencel cohort compared to the SOC cohort ($p = 0.762$).

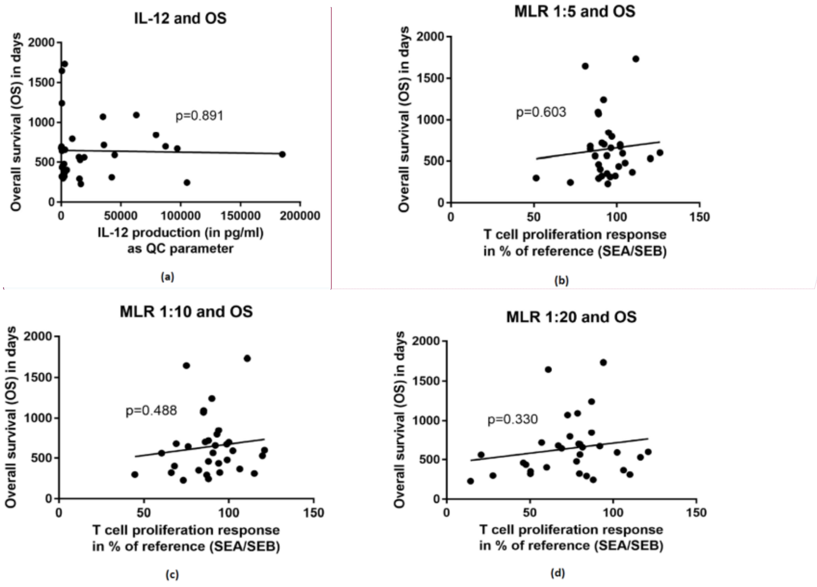

Commented [M2]: "ml" in figure s2a should be changed to "mL"

Commented [FE3R2]: I would rather prefer to have "ml" here

Figure S2. Relation of DC vaccine quality control and overall survival (OS). One main quality control parameter measured during the production of the personalized, autologous vaccine was the IL-12

production capacity of every single vaccine. When analyzing a possible correlation with OS, no such connection could be made (**a**, $p = 0.891$). Similarly, the T cell proliferation capacity of the DC vaccine did not have a connection to survival — for three different DC: T cell ratios tested: 1:5 (**b**),1:10 (**c**), 1:20 (**d**). Overall, vaccine quality did not have an influence on OS.

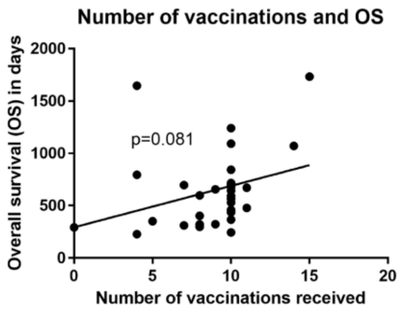

Figure S3. Analysis of potential influence of number of vaccinations received on overall survival (OS). In a Pearson correlation calculation, a non-significant trend towards better OS based on the number of vaccinations received can be registered ($p = 0.081$).

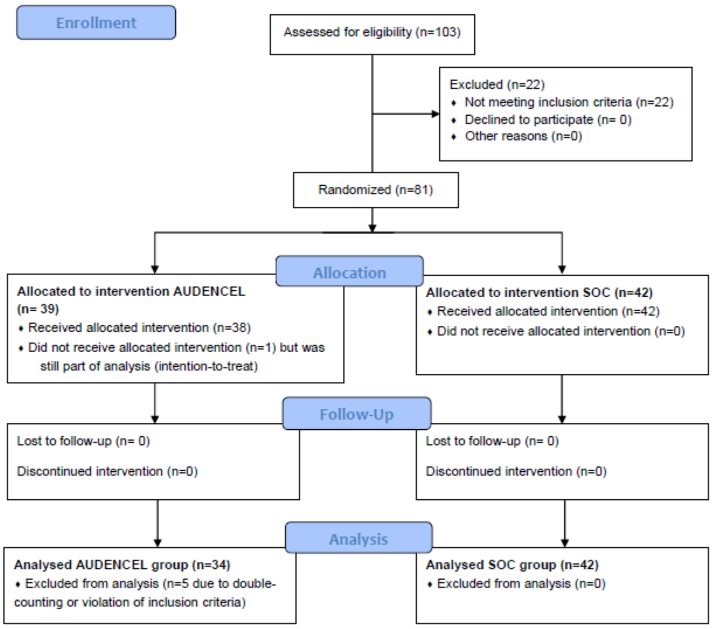

Figure S4. CONSORT 2010 Flow Diagram.

Table S1. CONSORT 2010 checklist of information to include when reporting a randomised trial *.

Section/Topic	Item No	Checklist item	Reported on page No
**Title and abstract**			
	1a	Identification as a randomised trial in the title	1
	1b	Structured summary of trial design, methods, results, and conclusions (for specific guidance see CONSORT for abstracts)	1–2
**Introduction**			
Background and objectives	2a	Scientific background and explanation of rationale	2–3
	2b	Specific objectives or hypotheses	2–3
**Methods**			
Trial design	3a	Description of trial design (such as parallel, factorial) including allocation ratio	2, 11–13
	3b	Important changes to methods after trial commencement (such as eligibility criteria), with reasons	3, 12
Participants	4a	Eligibility criteria for participants	11
	4b	Settings and locations where the data were collected	11
Interventions	5	The interventions for each group with sufficient details to allow replication, including how and when they were actually administered	11–13
Outcomes	6a	Completely defined pre-specified primary and secondary outcome measures, including how and when they were assessed	11–13
	6b	Any changes to trial outcomes after the trial commenced, with reasons	-
Sample size	7a	How sample size was determined	13
	7b	When applicable, explanation of any interim analyses and stopping guidelines	-
Randomisation:			
Sequence generation	8a	Method used to generate the random allocation sequence	11
	8b	Type of randomisation; details of any restriction (such as blocking and block size)	11
Allocation concealment mechanism	9	Mechanism used to implement the random allocation sequence (such as sequentially numbered containers), describing any steps taken to conceal the sequence until interventions were assigned	11
Implementation	10	Who generated the random allocation sequence, who enrolled participants, and who assigned participants to interventions	11–13
Blinding	11a	If done, who was blinded after assignment to interventions (for example, participants, care providers, those assessing outcomes) and how	- (open-label)
	11b	If relevant, description of the similarity of interventions	-
Statistical methods	12a	Statistical methods used to compare groups for primary and secondary outcomes	13
	12b	Methods for additional analyses, such as subgroup analyses and adjusted analyses	13

**Results**			
Participant flow (a diagram is strongly recommended)	13a	For each group, the numbers of participants who were randomly assigned, received intended treatment, and were analysed for the primary outcome	3–4
	13b	For each group, losses and exclusions after randomisation, together with reasons	3–4
Recruitment	14a	Dates defining the periods of recruitment and follow-up	11, 4
	14b	Why the trial ended or was stopped	11
Baseline data	15	A table showing baseline demographic and clinical characteristics for each group	3–4
Numbers analysed	16	For each group, number of participants (denominator) included in each analysis and whether the analysis was by original assigned groups	3–4
Outcomes and estimation	17a	For each primary and secondary outcome, results for each group, and the estimated effect size and its precision (such as 95% confidence interval)	4–6
	17b	For binary outcomes, presentation of both absolute and relative effect sizes is recommended	4–6
Ancillary analyses	18	Results of any other analyses performed, including subgroup analyses and adjusted analyses, distinguishing pre-specified from exploratory	7–9
Harms	19	All important harms or unintended effects in each group (for specific guidance see CONSORT for harms)	6–7
**Discussion**			
Limitations	20	Trial limitations, addressing sources of potential bias, imprecision, and, if relevant, multiplicity of analyses	9–11
Generalisability	21	Generalisability (external validity, applicability) of the trial findings	9–11
Interpretation	22	Interpretation consistent with results, balancing benefits and harms, and considering other relevant evidence	9–11
**Other information**			
Registration	23	Registration number and name of trial registry	11
Protocol	24	Where the full trial protocol can be accessed, if available	11
Funding	25	Sources of funding and other support (such as supply of drugs), role of funders	13

* We strongly recommend reading this statement in conjunction with the CONSORT 2010 Explanation and Elaboration for important clarifications on all the items. If relevant, we also recommend reading CONSORT extensions for cluster randomised trials, non-inferiority and equivalence trials, non-pharmacological treatments, herbal interventions, and pragmatic trials. Additional extensions are forthcoming: for those and for up to date references relevant to this checklist, see www.consort-statement.org.

Supplementary Materials and Methods

Immunotherapy: Description of the Audencel DC vaccine

Audencel is a DC-based autologous cancer vaccine. It is comprised of DCs charged with tumor-derived antigens that are matured via LPS and IFNγ and that are characterized by the secretion of IL-12. After production, the final DC vaccine product is 1-5 x 10^6 autologous, "semi-mature" DCs in a DMSO-containing freezing medium (CryoStore CS2/Lite, STEMCELL Technologies, Vancouver, CA).

Immunotherapy: Prior studies on Audencel

The DC vaccine technology behind Audencel has been investigated in prior preclinical studies and in a phase I clinical trial [1–4]. Preclinically, Felzmann et al. showed in human *in vitro* experiments that maturation of DCs with LPS and IFNγ leads to an immunostimulatory phenotype (characterized by IL-12 secrection) that can efficiently triggered cytolytic activity in autologous T lymphocytes. Importantly, this was only the case for co-cultures performed 2–6 h after maturation stimulus (LPS/ IFNγ) but not for co-cultures performed at 48 hours [1]. The Audencel technology thus uses DCs matured for 6 hours, called "semi-mature". Hüttner et al. studied the DC generation technique used for Audencel in a syngeneic murine *in vivo* model and found that the IL-12 secreting DCs used could reduce tumor growth (murine cell line K-Balb) [2]. The method for generating DCs from peripheral blood monocytes was evaluated in further human *in vitro* experiments by Felzmann et al [3]. Finally, the Audencel technology was also tested in a phase I clinical trial where pediatric cancer patients suffering from advanced solid pediatric malignancies were vaccinated by Dohnal et al [4]. This study established feasibility and safety. As a continuation of these early preclinical and clinical experiences, the here presented phase II trial of Audencel applied to patients suffering from glioblastoma was initiated.

Immunotherapy: Production of the Audencel DC vaccine for the phase II clinical trial on glioblastoma

Tumor samples were harvested through surgical resection, irradiated with 12,000 rad (in accordance with local guidelines for the irradiation of blood products for human transfusion) and then stored subsequently without further delay at 4°C and transported to our Good Manufacturing Practice (GMP) facilities under sterile conditions for the generation of autologous tumor lysate. For that, tumor tissue was kept in Phosphate Buffered Saline (PBS; Hyclone, ThermoScientific, Utah, USA), was disrupted mechanically via a scalpel, pressed through a nylon mesh and the resulting cells in single cell suspension were lysed by five freeze/thaw cycles (liquid nitrogen, −150°C) in distilled water resulting in tumor cell lysate ready for further use. Particulate components were removed by centrifugation. Protein concentration of each tumor lysate was determined by Bradford assay and the vials containing protein lysate were kept frozen at −80°C.

Peripheral blood mononuclear cells (PBMCs) were obtained by leukocyte apheresis (performed at the Transfusion Medicine departments of the respective treatment centers according to local protocols and yielding 4–10 × 10^9 mononuclear cells) followed by elutration (Elutra cell separator, Gambro BCT, Inc. Lakewood, Colorado, USA) for the selective enrichment of clinical-scale monocytes. Then, monocytes were cultured *in vitro* in Cellgro medium (CellGenix Technology, Freiburg, Germany) with the presence of (317U/ml) recombinant human interleukin-4 (IL-4, CellGenix Technology, Freiburg, Germany) and (1000U/ml) recombinant human granulocyte macrophage-colony stimulating factor (rhGM-CSF, CellGenix Technology, Freiburg, Germany) at a density of 1x10^6 monocytes/cm^2. On day 3, fresh medium containing the same cytokines, at the same concentration was added. On day 6, immature dendritic cells were incubated with autologous tumor lysate (see above) together with the immunological adjuvant Keyhole Limpet Hemocyanin (KLH, Calbiochem, Darmstadt, Germany) for 2 hours prior maturation stimulus. Subsequently, the DCs were incubated with LPS (200U/ml, E. coli strain O111:B4, Calbiochem, San Diego, CA, USA) and IFNy (50ng/mL, Boehringer Ingelheim,Vienna, Austria) for 6 hours to induce functional maturation.

"Semi-mature" DCs were then harvested, washed with Phosphate Buffer Saline (PBS, Hyclone, ThermoScientific, Utah, USA), aliquoted to vials containing (1-)5 × 10^6 DCs each and stored in a liquid nitrogen tank.

Immunotherapy: Quality control

At the arrival of tumor material for vaccine production at the GMP facility, a sterility test was immediately conducted (BACTEC system, BD Biosciences, NJ, USA). Only if sterility could be proven, the material was processed further. Two aliquots of each final vaccine batch after production (see above) were again used for quality control that included tests for viruses, mycoplasma, and bacteria according to standard clinical guidelines. In addition, functional potency and the phenotype of the tumour lysate-loaded DCs was examined *in vitro*.

The purity and phenotype of each DC lot was determined by flow cytometry (FACS Calibur, Becton Dickinson, San Jose, CA). Cells were stained with antibodies against CD45 (BD PharMingen, San Diego, CA, USA), CD14 (BD PharMingen, San Diego, CA, USA), Major Histocompatibility Complex (MHC-I and –II, Dako Cytomation, Glostrup, Denmark), CD1a (BD PharMingen, San Diego, CA, USA), CD83 (BD PharMingen, San Diego, CA, USA), CD80 (Immunotech, Marseille, France) and CD86 (BD PharMingen, San Diego, CA, USA). Release criteria for usage in clinical application were more than 70% viable dendritic cells and more than 60% CD86+/MHCI+/MHCII+/CD80+/CD83+ expression on these cells.

To ensure functional potency prior to application to patients, IL-12 production capacity and T-cell stimulation capacity were determined for each batch of the DC vaccine. For IL-12 measurement, an ELISA test system was used. Briefly, one vial of the vaccine was thawed, and the cells were used 24 hours after thawing. 96-well plates were coated with capture antibody (BD, San Jose, CA) diluted in PBS with 0,02% sodium azide. The next day, unspecific binding in the wells was blocked with 2% BSA in PBS. After blocking, IL-12 standard solutions (BD, San Jose, CA) of known concentration (30-1250 pg/ml) and diluted samples (1:2; 1:20; 1:50) were distributed into the wells. On the third day, captured cytokines were detected by primary incubation with a biotinylated detection antibody (BD, San Jose, CA) and secondary via incubation with alkaline phosphatase-conjugated streptavidin (Chemicon, Temecula, C). When phosphatase substrate at a concentration of 1 mg/ml in diethanolamine buffer was added, the respective yellow colour reaction developed. The diethanolamine buffer consisted of 1 M diethanolamine and 0,5 mM MgCl2 diluted in sterile water with a pH of 9.8. The optical density was measured with an ELISA reader (Anthos, Salzburg, Austria) at a wavelength of 405 nm and a reference wavelength of 690 nm. The cytokine concentrations was calculated using the WinRead V.2.3 software. The release criterion for application of the batch for human use in the trial was >100pg/ml IL-12.

For measurement of T-cell stimulation capacity, allogeneic mixed leukocyte reactions (alloMLR) were carried out. Briefly, allogeneic responder peripheral blood mononuclear cells (PBMCs) collected from healthy donors were isolated by gradient centrifugation from peripheral blood and recovered in AIM-V medium (ThermoFisher, Waltham, MA) supplemented with 2% human plasma (Octapharm, Vienna, Austria). Stimulating DCs (10.000, 2.000, or 400) were placed in triplicates (100 µl per well) on a 96 well round-bottom plate and 10^5 responder cells in 100 µl medium were added to each well. For a positive reference 10^5 responder cells were stimulated in 100 µl medium with Staphylococcal enterotoxin A/B (SEA/SEB, Toxin Technologies Inc., Sarasota, FL) at 100 ng/ml final concentration. On day 4 of the co-culture, 1 µCi of tritium thymidine solution (NEN Life Science Products, Boston, MA) was added to each well and the cells were incubated for another 18 hours. Finally, the cells were harvested with a Skatron harvesting device (Skatron, Lier, Norway) and the incorporated tritium thymidine was counted on a Trilux β-plate reader (Wallac Oy, Turku, Finland). The release criterion for application of the batch for human use in the trial was a T-cell proliferation of at least 30% of the reference SEA/SEB response (for the DC:T-cell ratios 1:5 and 1:10 and at least 15% for the DC:T-cell ratio 1:20).

Summing up, all batches of the DC vaccine that were released to the patient had shown IL-12 production capacity and T-cell stimulation capacity as well as a pre-defined stimulatory phenotype

and absence of contamination with pathogens. After quality control, the personalized, autologous DC vaccine for each patient was then kept frozen until application. At the time of treatment an aliquot of the DC cancer vaccine containing approximately 1-5 million DCs was thawed and inoculated to the corresponding patient by ultrasound-guided injection intranodally into a tumor-free (cervical) lymph node.

Immunotherapy: Treatment schedule

All patients received the first line standard therapy for GBM: surgery, radiotherapy, and chemotherapy (Temozolomide). Randomization was done following surgery; patients in the treatment arm who received Audencel as an add-on to the standard treatment underwent leukocyte apheresis within 7-14 days after surgery. The first 4 immunizations were administered in weeks 7-10. Six more immunizations were applied in between the 6 blocks of maintenance chemotherapy. After completion of that schedule, patients received boost immunizations every 3 months. The vaccine was applied intranodally; each vaccine aliquot of Audencel contained 1-5 x 10^6 DC. The immunization schedule continued unaltered even if patients suffered disease recurrence and Temozolomide was withdrawn and replaced with an alternative therapy such as Bevacizumab. Patients of both groups received supportive care for acute or chronic toxicity whenever indicated.

References

1. Felzmann, T.; Hüttner, K. G.; Breuer, S. K.; Wimmer, D.; Ressmann, G.; Wagner, D.; Paul, P.; Lehner, M.; Heitger, A.; Holter, W. Semi-mature IL-12 secreting dendritic cells present exogenous antigen to trigger cytolytic immune responses. *Cancer Immunol. Immunother.* **2005**, *54*, 769–780.
2. Hüttner, K. G.; Breuer, S. K.; Paul, P.; Majdic, O.; Heitger, A.; Felzmann, T. Generation of potent anti-tumor immunity in mice by interleukin-12-secreting dendritic cells. *Cancer Immunol. Immunother.* **2005**, *54*, 67–77.
3. Felzmann, T.; Witt, V.; Wimmer, D.; Ressmann, G.; Wagner, D.; Paul, P.; Hiittner, K.; Fritsch, G. Monocyte enrichment from leukapharesis products for the generation of DCs by plastic adherence, or by positive or negative selection. *Cytotherapy* **2003**, *5*, 391–398.
4. Dohnal, A. M.; Witt, V.; Hügel, H.; Holter, W.; Gadner, H.; Felzmann, T. Phase I study of tumor Ag-loaded IL-12 secreting semi-mature DC for the treatment of pediatric cancer. *Cytotherapy* **2007**, *9*, 755–770.

© 2018 by the authors. Submitted for possible open access publication under the terms and conditions of the Creative Commons Attribution (CC BY) license (http://creativecommons.org/licenses/by/4.0/).
